# Conjuncted photo-thermoelectric effect in ZnO–graphene nanocomposite foam for self-powered simultaneous temperature and light sensing

**DOI:** 10.1038/s41598-020-68790-w

**Published:** 2020-07-17

**Authors:** Huiqi Zhao, Bangsen Ouyang, Lu Han, Yogendra Kumar Mishra, Zhiqiang Zhang, Ya Yang

**Affiliations:** 1 CAS Center for Excellence in Nanoscience, Beijing Key Laboratory of Micro-Nano Energy and Sensor, Beijing Institute of Nanoenergy and Nanosystems, Chinese Academy of Sciences, Beijing, 100083 People’s Republic of China; 2 School of Materials and Metallurgy, University of Science and Technology Liaoning, 185 Qianshan Zhong Road, Anshan, 114051 People’s Republic of China; 3 School of Nanoscience and Technology, University of Chinese Academy of Sciences, Beijing, 100049 People’s Republic of China; 4 Mads Clausen Institute, NanoSYD, University of Southern Denmark, Alsion 2, 6400 Sønderborg, Denmark; 5 School of Chemical Engineering, University of Science and Technology Liaoning, 185 Qianshan Zhong Road, Anshan, 114051 People’s Republic of China; 6 Center On Nanoenergy Research, School of Physical Science and Technology, Guangxi University, Nanning, 530004 Guangxi People’s Republic of China

**Keywords:** Sensors and biosensors, Porous materials

## Abstract

The self-powered sensors are more and more important in current society. However, detecting both light and temperature signals simultaneously without energy waste and signal interference is still a challenge. Here, we report a ZnO/graphene nanocomposite foam-based self-powered sensor, which can realize the simultaneous detection of light and temperature by using the conjuncted photo-thermoelectric effect in ZnO–graphene nanocomposite foam sensor. The output current under light, heating and cooling of the device with the best ZnO/graphene ratio (8:1) for the foam can reach 1.75 µA, 1.02 µA and 0.70 µA, respectively, which are approximately three fold higher than them of devices with other ZnO/graphene ratios. The ZnO–graphene nanocomposite foam device also possesses excellent thermoelectric and photoelectric performances for conjuncted lighting and heating detection without mutual interference. The ZnO–graphene nanocomposite foam device exhibits a new designation on the road towards the fabrication of low cost and one-circuit-based multifunction sensors and systems.

Notably, light and heat are the ubiquitous energy resources in daily life, but only a fraction of it is really utilized and the rest, a very large fraction is in general lost in the environment. This is mainly due to the fact that existing energy harvesting technologies are lacking with desired performances and efficiency and therefore the development of appropriate nanomaterials based energy harvesting technologies has been of prime interest for advanced materials and energy materials communities^1–4^. Significant attempts have been continued in this context, especially on the self-powered sensors, which have the ability to scavenge the energies from ambient environmental stimuli, such as light, heat, etc. and can convert these stimulis into electricity^5–7^. However, the simultaneous and sensitive detection of light and temperature with a single device is still an open issue to be solved, because most of the sensors can only effectively detect an individual source of signal, leading to high cost and low power conversion efficiency^8^. Despite of the fact that researchers have developed the dual-parameter temperature–pressure-sensing devices, these devices are mainly based on organic materials still. Moreover, the muctual influence between both the stimuli signals and complicated device fabrication process still hamper the development of multifunctional sensors with desired high efficiency^9–15^. However, to the best of our literature knowledge, there exists no such report about the conjuncted photo-thermoelectric effect in self-powered sensor device. It is necessary to develop a multifunctional and user friendly sensor device which can detect both the signals, i.e., light and temperature, simultaneously without energy waste and signal interference.

Material involved in the sensor device is the most important candidate as it’s functionality is mainly going to decide the sensitivity, selectivity, efficiency, etc. hence the choice of the material is a very crucial factor in device fabrication. From the broad metal oxide materials family, zinc oxide (ZnO) has attracted extensive interest owing to its unique hexagonal-wurtzite crystal structure, wide direct bandgap (~ 3.37 eV) and high exciton binding energy (~ 60 meV), etc^10,16–23^. Specially, one dimensional (1D) ZnO nanowires^19,22,24–28^ and tetrapods (T-ZnO)^16, 17,20^ nano materials with excellent photoelectric properties have been widely used for photo-detection^29–32^. Definitely, ZnO is very good material candidate in terms of light sensing but from the thermal conductivity point of view, it exhibits a poor response, especially, in environmental temperature range and therefore to enhance the temperature sensitivity another material component which has a high electrical conductivity, is desired as complementary material in the device. In this context, graphene exhibits exceptional electrical properties, thermal conductivity and mechanical properties, including high carrier mobility, large specific surface area, high crystal quality and flexible sp^2^ hybridization structure^33–37^, and it can be effectively applied to enhance the performances of photo-thermal and mechanical energy conversion devices, especially thermoelectric sensors^36,38–40^. Some studies have investigated a graphene-based pyroelectric bolometer for detecting the warm bodies in their proximity^38^, and a pyroelectric infrared (IR) sensor with graphene electrodes can improve the operating frequency instead of conventional electrode^39^. Recently, some researchers have reported that graphene-based terahertz photodetectors, whose performance can be dramatically improved by photo-thermoelectric effect (PTE)^41,42^. However, there are few studies on multifunctional self-powered photodetector which can detect and distinguish light energy and heat energy at the same time. Meanwhile, during the fabrication of multi-nanomaterial components-based sensor device, the homogeneity is often a very big issue because of self-agglomeration behaviour of individual nano components. This is nearly impossible for identical nanomaterials and hence involved materials in appropriate forms need to be conjugated. Shape of the nanostructure could play very important role in this context, especially tetrapodal structures from ZnO which are basically constituted via 4 interconnected 1D ZnO rods via a central core. Tetrapod shape offers a high advantage in the sense that does not matter how are they placed with respect to each other, they constitute a self-assembled homogeneous 3D network with extremely high porosity where the other material component can be easily incorporated with high homogeneity. In such a material form, both the component are homogeneously distributed throughout the entire material^20^ and this can be used to build a reliable and portable devices for example, the conveniently-manufacture sensor device based on graphene and tetrapod ZnO composite materials is demonstrated here for the first time to the best of literature knowledge.

In this work, we report a ZnO/graphene nanocomposite foam-based sensor, which can simultaneously harvest and convert thermal/light energies through the ZnO–graphene nanocomposite foam for self-powered sensing. The best ZnO/graphene ratio for the foam device is 8:1, which exhibits excellent photoelectric and thermoelectric performances. As a result, the photoelectric response can reach 1.75 µA (247.8 mW/cm^2^), the heating response is about 1.02 µA (17.9 K) and the cooling response is 0.70 µA (− 10.5 K), which are approximately threefold higher than the devices with other ZnO/graphene ratios. From the temperature sensitivity and the response/ recovery time, it can be confirmed that the fabricated ZnO/graphene foam-based device exhibits excellent thermoelectric performances. The photoelectric sensitivity, responsivity (R), EQE, Detectivity (D*) and the response/recovery time indicate that the foam device can be a stable and high-performance photoelectric sensor. Moreover, it is important to note that the signals of ZnO–graphene nanocomposite foam device under conjuncted lighting and heating conductions can be detected without mutual interference. In other words, the foam device can implement that the light illumination does not interfere with the foam device when the temperature is detected. Moreover, temperature variation does not affect the detection of light intensity by using such a foam device. Therefore, the demonstrated ZnO–graphene nanocomposite foam device offers the realization of simultaneous detection of temperature and light through conjuncted photo-thermoelectric effect.

## Results and discussion

**Figure 1:**
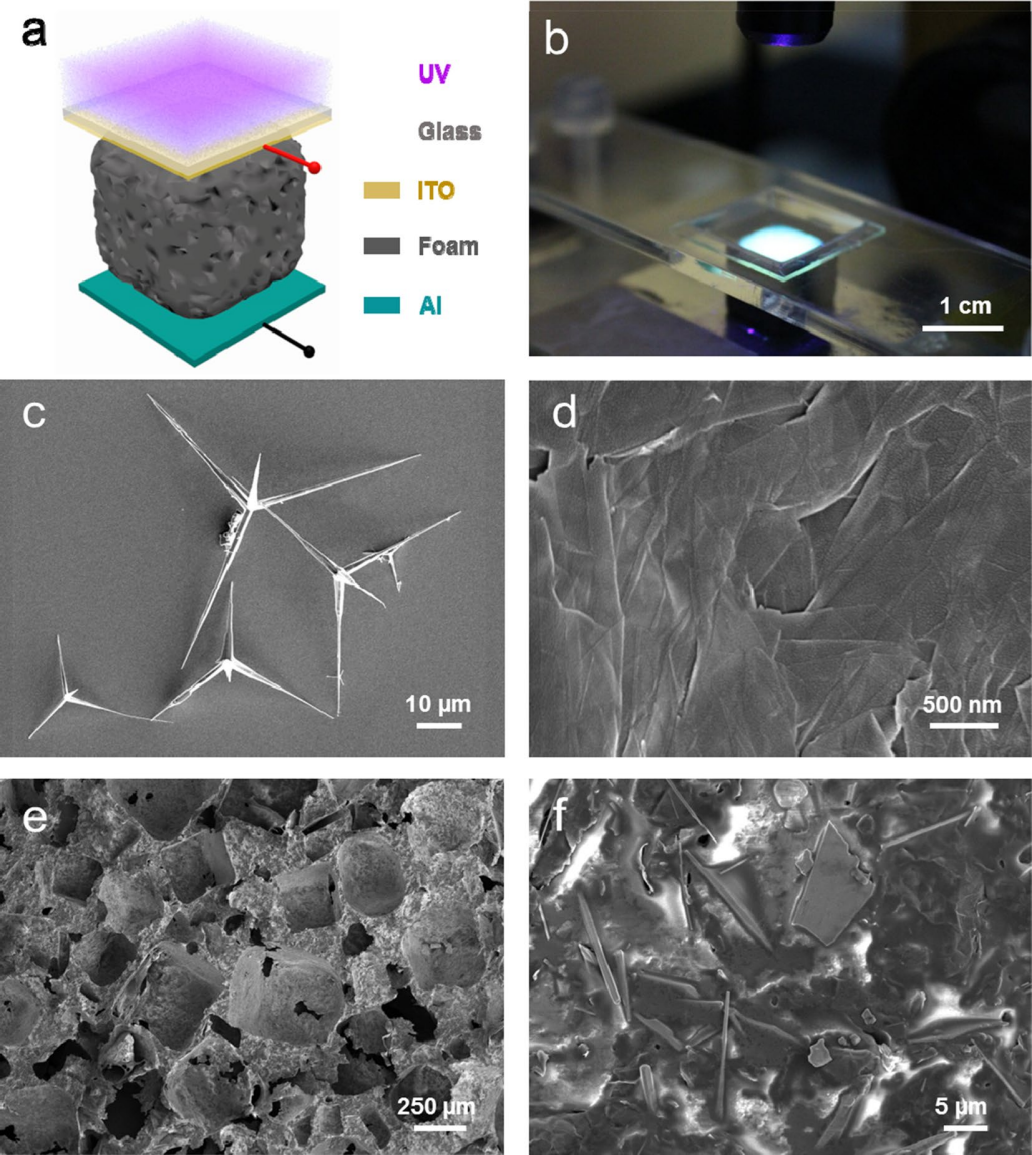
Structure and characterization of ZnO–graphene nanocomposite foam. (**a**) Schematic diagram of a ZnO–graphene nanocomposite foam device. (**b**) Photograph of the foam device under UV illumination. (**c**) SEM image of the tetrapod-like ZnO. (**d**) SEM image of graphene. (**e**) SEM image of the optimized foam (8:1) at low magnification. (**f**) SEM image of the optimized foam (8:1) at high magnification.

Figure 1a displays the structure diagram of the ZnO–graphene nanocomposite foam device for self-powered simultaneous temperature and light sensing. The sensor is mainly composed of a ZnO–graphene composite foam with an ITO electrode on the top surface and an Al electrode on the bottom surface. One also knows that the as-fabricated ZnO–graphene nanocomposite foam has porous structures synthesized by a sacrificial template method^43,44^. Ultraviolet light is irradiated from the top onto the composite foam device through the glass substrate. Figure 1b displays the photograph of the foam device when it is illuminated by ultraviolet (UV) light. As illustrated in Fig. 1b, the foam is approximately 1.0 cm × 1.0 cm × 1.0 cm in size. The tetrapod-like spatial three-dimensional structure of the T-ZnO from SEM images, which has tetrapod nanorod with a diameter about 1–2 µm at their base and a tip diameter of 100 nm are shown in Fig. 1c (Supplementary information Fig. S2a). The lengths of the tetrapod arms are in the range 15 ~ 40 µm. The particular shapes of T-ZnO exhibits a 3D geometry structure (four 1D nanoarms interconnected together via a central core), which can prevent agglomeration of the nanomaterial itself, and can benefit the stable formation of highly porous, flexible networks^17,20^. The X-ray diffraction (XRD) patterns of T-ZnO powders are shown in Supplementary information Fig. S2c, exhibiting a typical hexagonal-wurtzite crystal structure (PDF # 65-3411) with high crystallinity due to the diffraction peaks are narrow and strong^5,17,20^. Figure 1d shows the SEM image of graphene indicating a thin layer structure. The SEM images of the nanocomposite foam device at low magnification and high magnification are illustrated in Fig. 1e, f, respectively. The microscopic porous structure of the foam can be observed in Fig. 1e. Meanwhile, a good combination of the T-ZnO nanorod and the graphene sheet structure is exhibited in Fig. 1f at high magnification. The SEM images of other materials without T-ZnO and graphene blend are depicted in Supplementary information Fig. S1.

**Figure 2:**
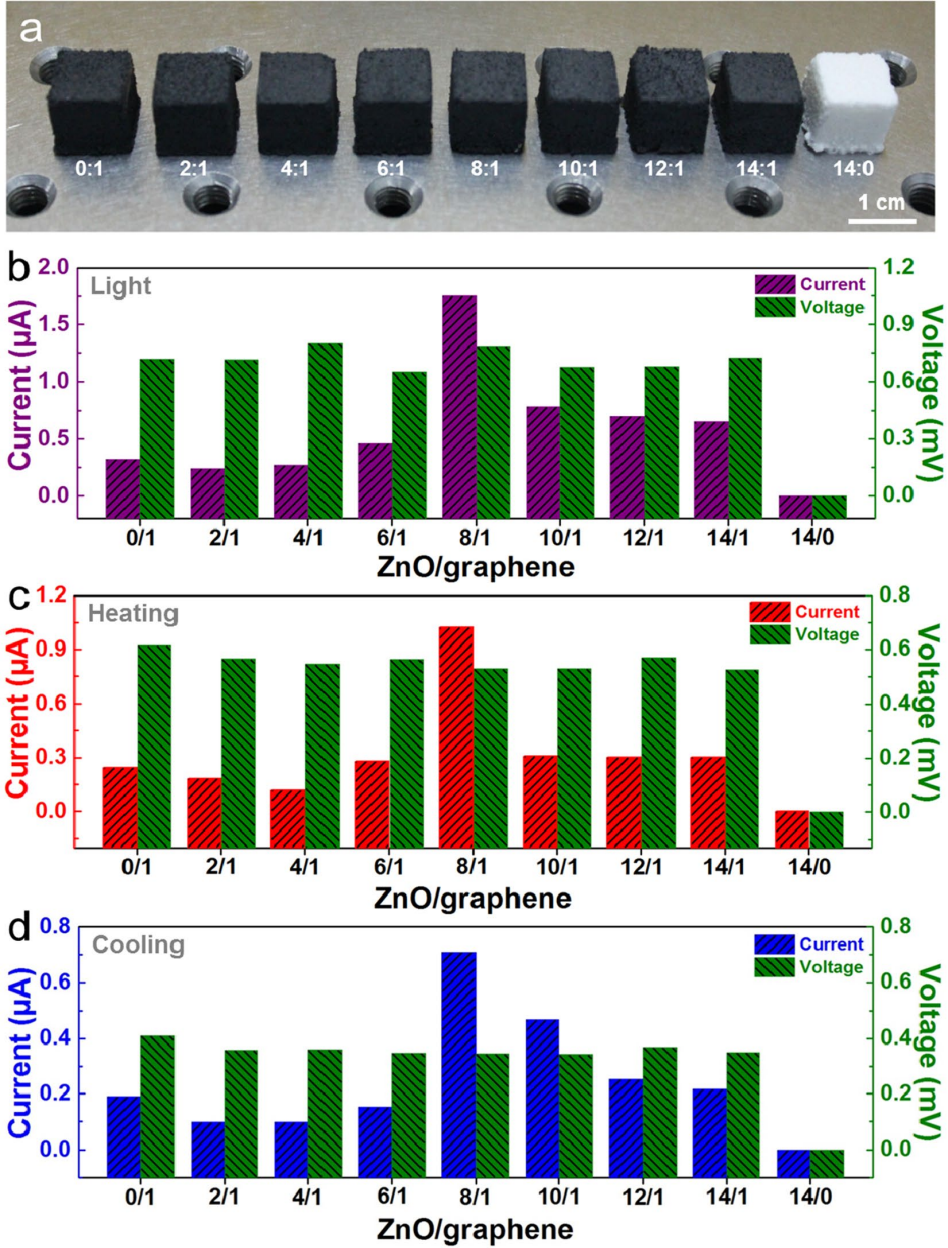
Performance statistics of the foam devices with different ZnO/graphene proportions. (**a**) Photograph of those foams with different ZnO/graphene ratio. (**b**–**d**) Output performance of different ZnO/graphene ratio devices under UV illumination (b), heating(c) and cooling (d) condition.

As illustrated in Fig. 2a, foam devices with different mass ratios of ZnO and graphene (ZnO/graphene = 0:1, 2:1, 4:1, 6:1, 8:1, 10:1, 12:1, 14:1, 14:0) were prepared to optimize the photoelectric and thermoelectric sensing performance. We measured the output performances of those foam devices under 365 nm wavelength light illumination (247.8 mW/cm^2^), heating and cooling. During testing the photoelectric performance of the foam device, the same light intensity is controlled by fixing the height and range of the light source. When testing the thermoelectric performance of the foam device, the same temperature is determined by the voltage applied to the Peltier cooler. The histogram of Fig. 2b–d are the average values of the current and voltage signals for different ZnO/graphene ratio foam devices, and the original test data is depicted in Supplementary information Fig. S3. Due to the output signals of the foam devices, they are basically identical in three cycles of continuous testing, indicating the sensing performance of the foam devices are stable (Supplementary information Fig. S3). Interestingly, as the proportion of ZnO/graphene increases, the current signal increases initially and then decreases, where the maximum current signal can be found at the ratio of 8:1. As demonstrated in Fig. 2b–d, the output current of the 8:1 foam device under light, heating and cooling are 1.75 µA, 1.02 µA and 0.70 µA respectively, which are higher than other devices by approximately three times. This is consistent with the I–V curves of foam devices with different ZnO/graphene ratios (Supplementary information Fig. S2b), indicating the best conductivity of the 8:1 device. Moreover, there is no difference in the output voltage of foam devices with different ZnO/graphene ratios, except for the device with a ratio of 14:0. The output current and voltage signals of the device with a ratio of 14:0 are both 0, which indicates the importance of graphene to the device. Therefore, the best sensing performance for a foam device with a ZnO/graphene ratio of 8:1 was optimized. The ZT value of the composite nanomaterials is considered to be the main reason for the excellent performance of the foam device, and the intrinsic physical mechanism is elaborated from the three parameters of the conductivity, thermal conductivity and Seebeck coefficient. Firstly, T-ZnO is a semiconducting material, which limits the electrical conductivity of pure T-ZnO device. The high proportion of ZnO leads to the large resistance of the foam device, which hinders the sensing. Owing to the fascinating electrical conductivity, the graphene plays a vital role in the foam device. However, the agglomeration effect of the pure graphene or the excessive graphene leads to the weak conductivity of devices. Hence, the addition of ZnO in proper proportion can separate the graphene sheets well and prevent agglomeration effect of the graphene itself, because of the unique three-dimensional structure of T-ZnO. From Supplementary information Fig. S2b, the conductivity of the ZnO/graphene (8:1) nanocomposite foam has been further improved. Secondly, the thermal conductivity of T-ZnO is about 40–55 Wm^−1^ K^−1^ at room temperature^45–47^. And the graphene exhibits a high thermal conductivity of 2000–5,000 Wm^−1^ K^−1^ at room temperature, which is higher than most of known materials^48–51^. But the excessive addition of graphene in composite foam will make it difficult to maintain the temperature difference of the foam device, resulting in little current and voltage. Thirdly, the thermoelectric performance of a device also can be evaluated via the Seebeck coefficient. At a room temperature, the Seebeck coefficient of ZnO and graphene is about − 300 to − 500 μV/K^46,47^ and 20 to 60 μV/K^41,51,52^, respectively. Thus, T-ZnO can be used for increasing the Seebeck coefficient of the foam device, thereby improving the thermoelectric properties of the devices. In a word, the combination of ZnO and graphene at appropriate ratios is considered a key factor for the promising performance of the foam device. Subsequently, the photo-thermoelectric sensing performance of the optimized foam device has been explored in detail.

**Figure 3:**
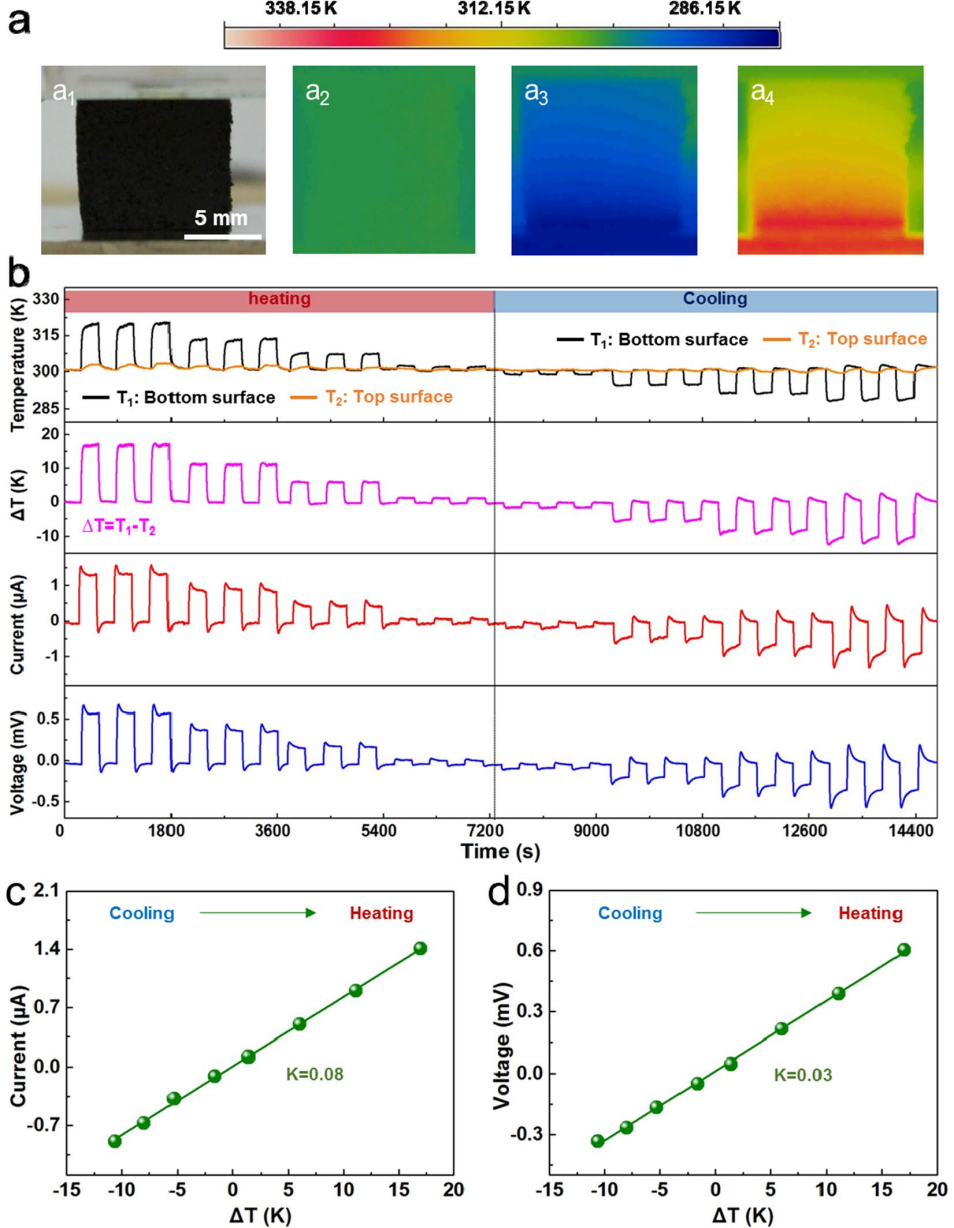
Thermoelectric properties of the optimized foam devices. (**a**) Photograph and infrared thermal images of the optimized foam device. (**b**) Output performance of the device at different temperatures. (**c**, **d**) Dependence of short circuit current (**d**) and output voltage (**d**) on ΔT.

The thermoelectric performances of the ZnO–graphene nanocomposite foam device are illustrated in Fig. 3. Figure 3a exhibits the photograph (a_1_) and infrared thermal images of the optimized foam device indicating the temperature distribution of the foam device at room temperature (a_2_), cooling (a_3_) and heating (a_4_). Under different temperature conditions (heating is on the left half part of Fig. 3b and cooling is on the right), the top and bottom surface temperature, ΔT, current signals and voltage signals of the optimized foam device are displayed in Fig. 3b. As the ΔT decreases, the output current and voltage of the foam device decrease proportionally. Correspondingly, as ΔT decreases from 17.9 to − 10.5 K, the average value of the steady current decreases from 1.4 to − 0.9 µA, and the average value of the steady voltage decreases from 0.6 to − 0.35 mV. As depicted in Fig. 3c, d, the output current and voltage increase linearly with ΔT with linearly fitted slopes of 0.08 and 0.03, respectively. In order to reflect the response speed, the rise time (the photocurrent increases from 10 to 90% of maximum value when light is switched on) and fall time (the photocurrent falls from 90 to 10% of the maximum value when light is switched off) are used^53^. From the viewpoint of thermoelectric performance, the response / recovery time of the foam device was found to be ~ 12 s/5 s for cooling (Supplementary information Fig. S4a, b) and ~ 15 s/9.5 s for heating (Supplementary information Fig. S4c, d).

**Figure 4:**
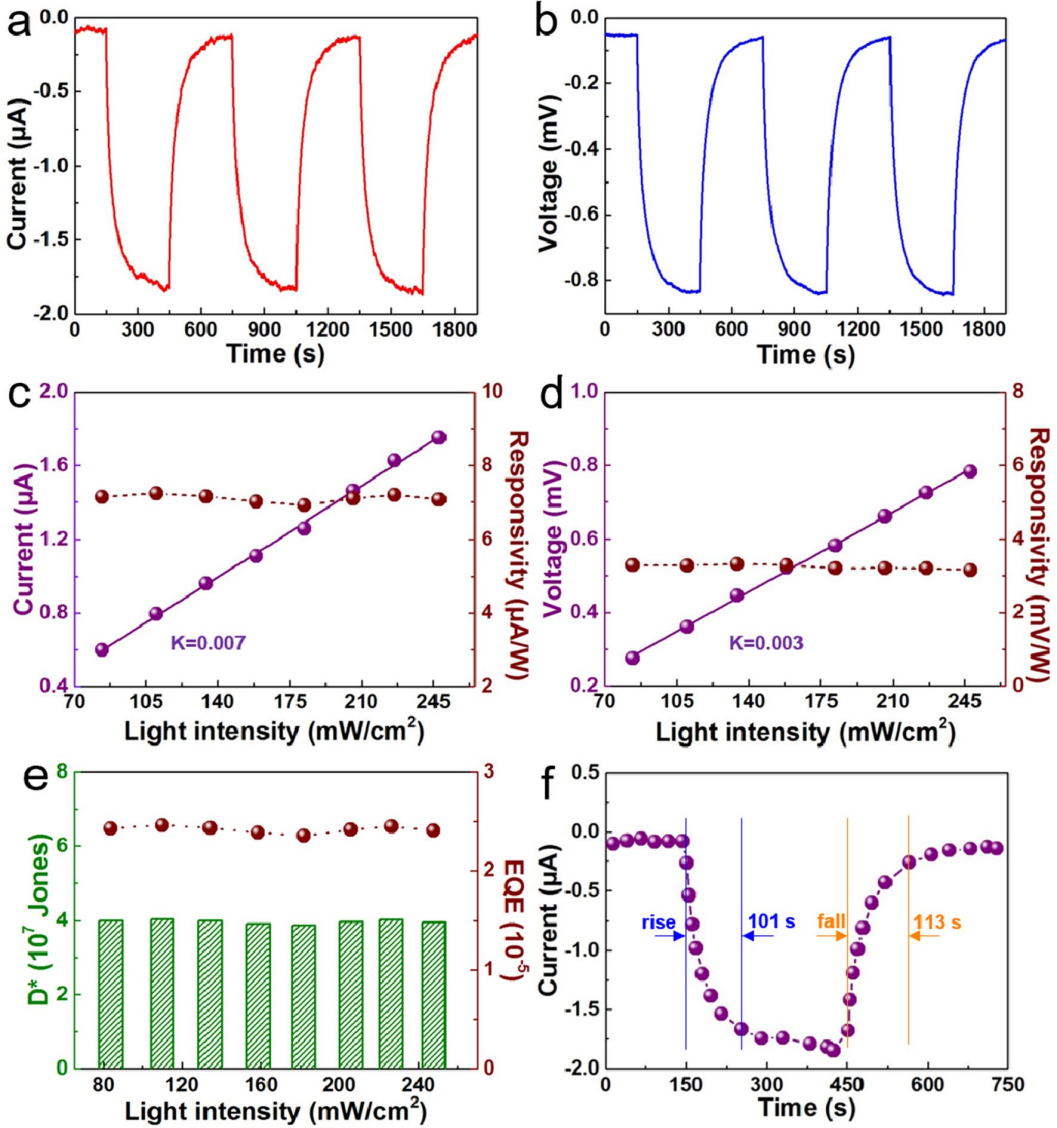
Photoelectric properties of the optimized foam devices. (**a, b**) Short circuit current (a) and (b) output voltage of the device under 365 nm UV illumination. (**c**) Short circuit current and responsivity (R) under different light intensities. (**d**) Output voltage and responsivity at different light intensities. (**e**) Detectivity (D*) and external quantum efficiency (EQE) at different light intensities. (**f**) Response and recovery time of the device under 365 nm light illumination.

Figure 4 demonstrates and analyzes the photoelectric performances of the ZnO–graphene nanocomposite foam device. The output current and voltage signals of the optimized device under periodical 365 nm UV illumination (247.8 mW/cm^2^) are illustrated in Fig. 4a, b, and we have also investigated the different output performances of the optimized device when the light intensity was changed from 226.2 to 83.6 mW/cm^2^. Under cyclic light illumination, the optimized foam device produces the same photoelectric signal substantially, proving that the photoelectric performance of the device is stable (Supplementary information Fig. S5). Meanwhile, as the light intensity decreases, the output signals of the foam device decrease proportionally. It is generally believed that high-performance photoelectric sensor should conform to the “5 s” requirements, because these requirements are closely related to the inherent properties of active materials and structures employed in the foam device^53^. Therefore, here we have summarized those requirements of the optimized foam device in Fig. 4c–f. As depicted in Fig. 4c, the fitted straight line with a slope of 0.007 indicates that the sensitivity is about 0.007 (mA cm^2^)/W. In order to eliminate the influences of incident light intensity and effective illumination area^53,54^, the responsivity (*R*) can be calculated by the following formula (Eq. 1)

1 $$R = \frac{{I_{{{\text{ph}}}} }}{PS}$$

where *I*_ph_ is the photocurrent of a foam device, *P* is the incident light intensity, and *S* is the effective illumination area.

The responsivity of photocurrent (Fig. 4c) and photovoltage (Fig. 4d) is basically identical under different light intensities. As displayed in Fig. 4d, the fitted straight line with a slope of 0.003 indicates that a sensitivity of 0.003 (V cm^2^)/W and excellent linearity too. The external quantum efficiency (EQE) estimation reflects the energy conversion efficiency from light to electric^53^, and it is an important parameter of the foam device which is calculated as follows (Eq. 2)

2 $${\text{EQE}} = \frac{{R_{\lambda } hc}}{e\lambda } \times 100\%$$

Here *R*_λ_ is the calculated responsivity at certain wavelength, *h* is the Planck constant, and *c* is the speed of light, *e* is the electron charge, *λ* is the wavelength of incident light.

Detectivity (D*) represent the minimum detectable power of irradiation, which can indicate the sensitivity of the device under weak light signal^53,55^. It can reflect the noise signal of the device and can be denoted as

3 $$D^{ * } = \frac{{R_{\lambda } }}{{\left( {2eJ_{{\text{d}}} } \right)^{1/2} }}$$

where *e* is the electron charge and *J*_d_ is the dark current density. As illustrated in Fig. 4e, the values of EQE and D* for ZnO–graphene nanocomposite foam device at different light intensities are approximately ~ 2.5 × 10^−5^ and ~ 4 × 10^7^, respectively. The next parameter to judge the photoelectric performance of the foam device is the response time which is obtained to be around ~ 101 s and the recovery time of the foam device is ~ 113 s under 365 nm UV light (Fig. 4f).

**Figure 5:**
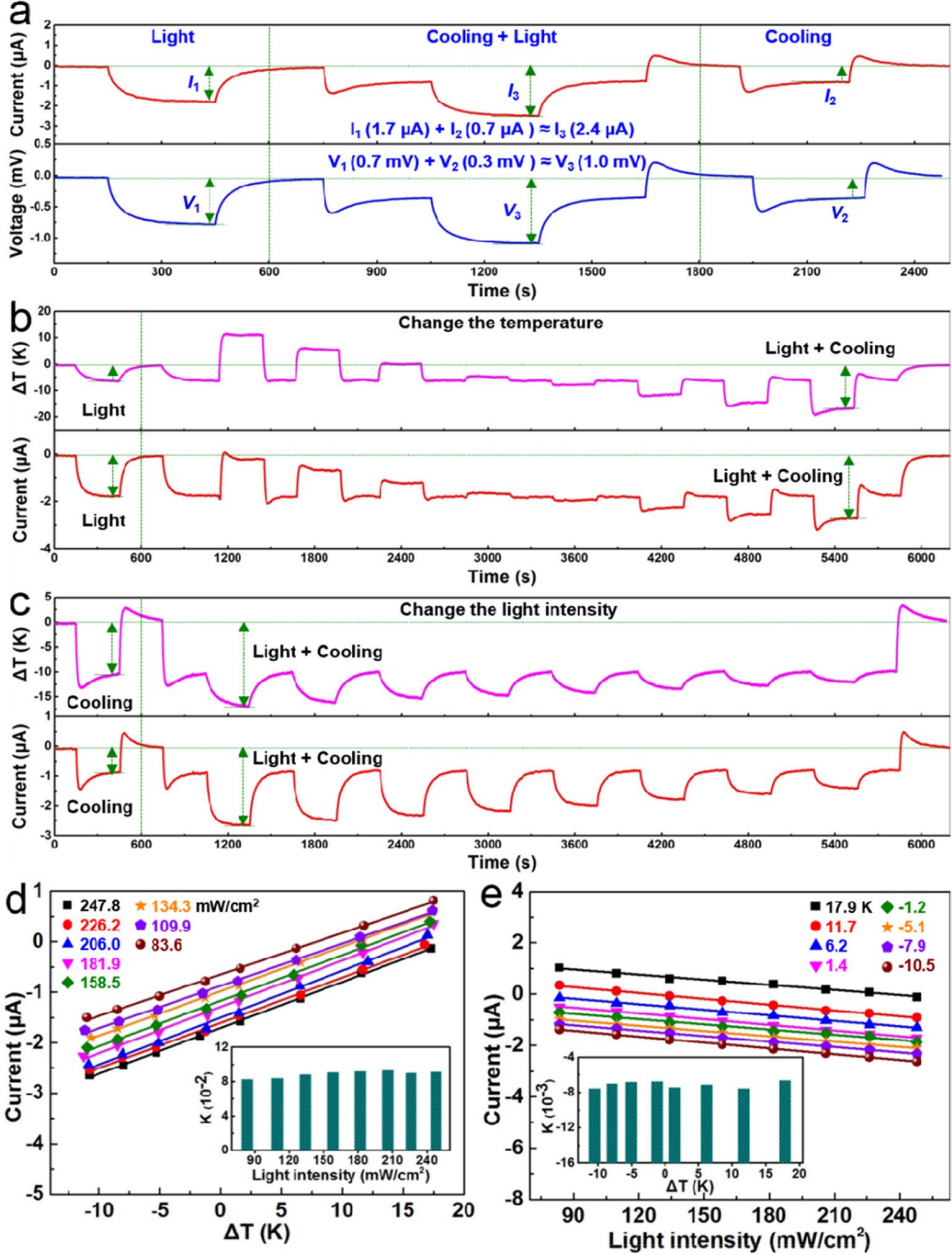
Photo-thermoelectric properties of the foam device. (**a**) Output current and voltage of the device under cooling, cooling + light, and light condition. (**b**) Photo-thermoelectric performance of the device at various temperature gradients when the light intensity is fixed at 247.8mW/cm^2^. (**c**) Photo-thermoelectric performance of the device under different light intensities when the ΔT is fixed at − 10.5 K. (**d**) Dependence of short circuit current on ΔT under different light intensities (247.8 to 83.6 mW/cm^2^). (**e**) Dependence of short circuit current on light intensity at different ΔT (− 10.5 to 17.9 °C).

Figure 5 shows the output performance of the ZnO–graphene nanocomposite foam-based device under the simultaneous stimulation of cooling (heating) at the bottom surface and light illumination at the top surface to explore the photo-thermoelectric effect in self-powered system. The current–time and voltage–time curves of the device induced by the light illumination, light illumination with cooling, and only cooling are illustrated in Fig. 5a (additional information is given in Supplementary information Fig. S6a). The corresponding peak current (I_1_) reached to 1.7 µA under 365 nm light illumination and the corresponding thermoelectric current peak (I_2_) rose to 0.7 µA under cooling condition. Interesting, we found the peak current (I_3_) reaching to 2.4 µA under lighting with cooling condition applied to the foam device. Therefore, the sum of I_1_ and I_2_ are equal to the output current under “lighting + cooling” conditions (I_3_). The corresponding peak voltage (V_1_) is 0.7 mV under 365 nm light illumination and the output voltage (V_2_) is 0.3 mV under cooling condition. Under both, when lighting and cooling were applied to the foam device, the peak voltage (V_3_) is about 1 mV. Therefore, the sum of V_1_ and V_2_ are equal to the output voltage under “lighting + cooling” conditions (V_3_). Meanwhile, the output signals of the device induced by the simultaneous lighting and heating are displayed in Supplementary information Fig. S6b which again proves the relationship among the output signals under lighting, heating and “lighting + heating” condition. It is also found that the sum of I_1_ (1.78 µA) and I_2_ (− 1.34µA) is about equal to I_3_ (0.4µA), and the sum of V_1_ (0.77 mV) and V_2_ (− 0.54 mV) are approximately equal to V_3_ (0.24 mV). Hence, these results indicate that the signals of ZnO–graphene nanocomposite foam-based device from lighting and heating exhibits no mutual interference. As illustrated in Fig. 5b, we also measured the current–time curves of the foam device under various temperature gradients when the light intensity is fixed at 247.8 mW/cm^2^, and the identical curves under different light intensities (from 247.8 to 83.6 mW/cm^2^) are observed as shown in Supplementary information Fig. S7. At a certain light intensity, the short circuit current increases near linearly with the increase of ΔT; besides, those fitted lines under different light intensities almost parallel to each other (Fig. 5d). The inset in Fig. 5d further suggests that temperature sensitivity is not affected by light intensity. Thus, the device can be successfully utilized to monitor the temperature variation and the light illumination simultaneously. As shown in Fig. 5c, we measured the current–time curves of the foam device under various light intensities under constant ΔT fixed at − 10.5 K, and the similar curves under different ΔT (− 10.5 to 17.9 K) are demonstrated in Supplementary information Fig. S8. The light sensing characteristics of the foam device under different temperature differences (− 10.5 to 17.9 K) are systematically studied in Fig. 5e. The fitted lines in Fig. 5e possess similar slope (inset in Fig. 5e) indicates that photoelectric signal is not influenced by ΔT. The foam device can be applied for light sensing upon various temperature differences. Therefore, it is obvious that the ZnO–graphene nanocomposite foam can achieve temperature and light sensing simultaneously without signal interference.

## Conclusion

In summary, a ZnO/graphene nanocomposite foam with conjuncted photo-thermoelectric effect has been utilized for efficient self-powered sensing of temperature and light signals. The best ZnO/graphene ratio for the foam device is 8:1, which has excellent photoelectric and thermoelectric performances. The photoelectric response reaches to 1.75 µA (247.8 mW/cm^2^), while the heating response is ~ 1.02 µA (17.9 K) and the cooling response is ~ 0.70 µA (− 10.5 K). The observed photoelectric performance parameters, responsivity, EQE, Detectivity (D*) and the response/recovery time indicate that the foam device is a stable and high-performance photoelectric sensor. Moreover, it can be confirmed that the sensing signals of ZnO/graphene nanocomposite foam device from conjuncted lighting and heating (cooling) conditions have no mutual interference. The ZnO–graphene nanocomposite foam-based devices is able to simultaneously achieve temperature and light sensing by conjuncted photo-thermoelectric effect which can be easily employed to build the cost-effective advanced energy harvesting technologies for sustainable future with high reliability and performance.

## Methods

### Fabrication of ZnO/graphene nanocomposite foam

Composite material mixing: Washing the stir bar and beaker required for the experiment, and dry it for use. Firstly, Prepolymer (2.0 g) and crosslinker (0.2 g) of PDMS elastomer (DC 184, Dow-Corning Corporation) were mixed in a weight ratio of 10:1, and stir quickly to uniform mixing. ZnO tetrapods were purchased from a company (Chengdu Jiaoda Jingyu Technology Co., Ltd) in China. Secondly, add tetrapod ZnO and graphene (the ratio of ZnO/graphene is 0:1, 2:1, 4:1, 6:1, 8:1, 10:1, 12:1, 14:1, 14:0) and stir evenly, the specific addition amount of T-ZnO and graphene is displayed in Supplementary information Table S1. Finally, NaCl powder (9 g) was added to the composite materials, and stirred well for about 30 min. Curing molding: The uniformly mixed composite material was added to the acrylic mould (1 cm × 1 cm × 1 cm) cut by the laser cutting machine, pressed by a homemade pressing rod. Then, the prepared cubic foams were placed in a preheated 90 °C oven, and solidified for 3 h. Pore-forming: The cured composite material was immersed in deionized water for 48 h, and the deionized water was periodically replaced (about 4 h). After the immersion was completed, those foams were taken out and dried in an oven.

### Characterization and measurements

The nanostructures of the foam, tetrapod ZnO and graphene were observed by scanning electron microscopy (Hitachi SUS8020). The ZnO powder were characterized by X-ray diffraction with Cu Kα radiation (XRD, PANalytical X’Pert3 Powder). A semiconductor thermoelectric cooling system was utilized to supply temperature variations. An infrared thermal imaging device was used to record the temperature variations of the ZnO–graphene nanocomposite foam-based device. A 405 nm light-emitting diode (LED) was used as the light source, and the light power density was detected using a calibrated power meter (OPHIR Starlite). Both the output current and output voltage signals of the nanogenerator were measured by a Keithley 2611B system sourcemeter.

## Supplementary information

Supplementary information
