# Supplementary material for: Conjuncted photo-thermoelectric effect in ZnO–graphene nanocomposite foam for self-powered simultaneous temperature and light sensing
Source: Sci Rep. 2020 Jul 17;10:11864. doi: 10.1038/s41598-020-68790-w (PMC7368035; doi:10.1038/s41598-020-68790-w)
Supplement: Supplementary file 1 — Supplementary information [file 41598_2020_68790_MOESM1_ESM.docx]

Supplementary for

**Conjuncted photo-thermoelectric effect in ZnO-graphene nanocomposite foam for self-powered simultaneous temperature and light sensing**

**Huiqi Zhao,^1,3^ Bangsen Ouyang,^1,3^ Lu Han^*2^ Yogendra Kumar Mishra,^4^ Zhiqiang Zhang,^5^ and Ya Yang^*1,3,6^**


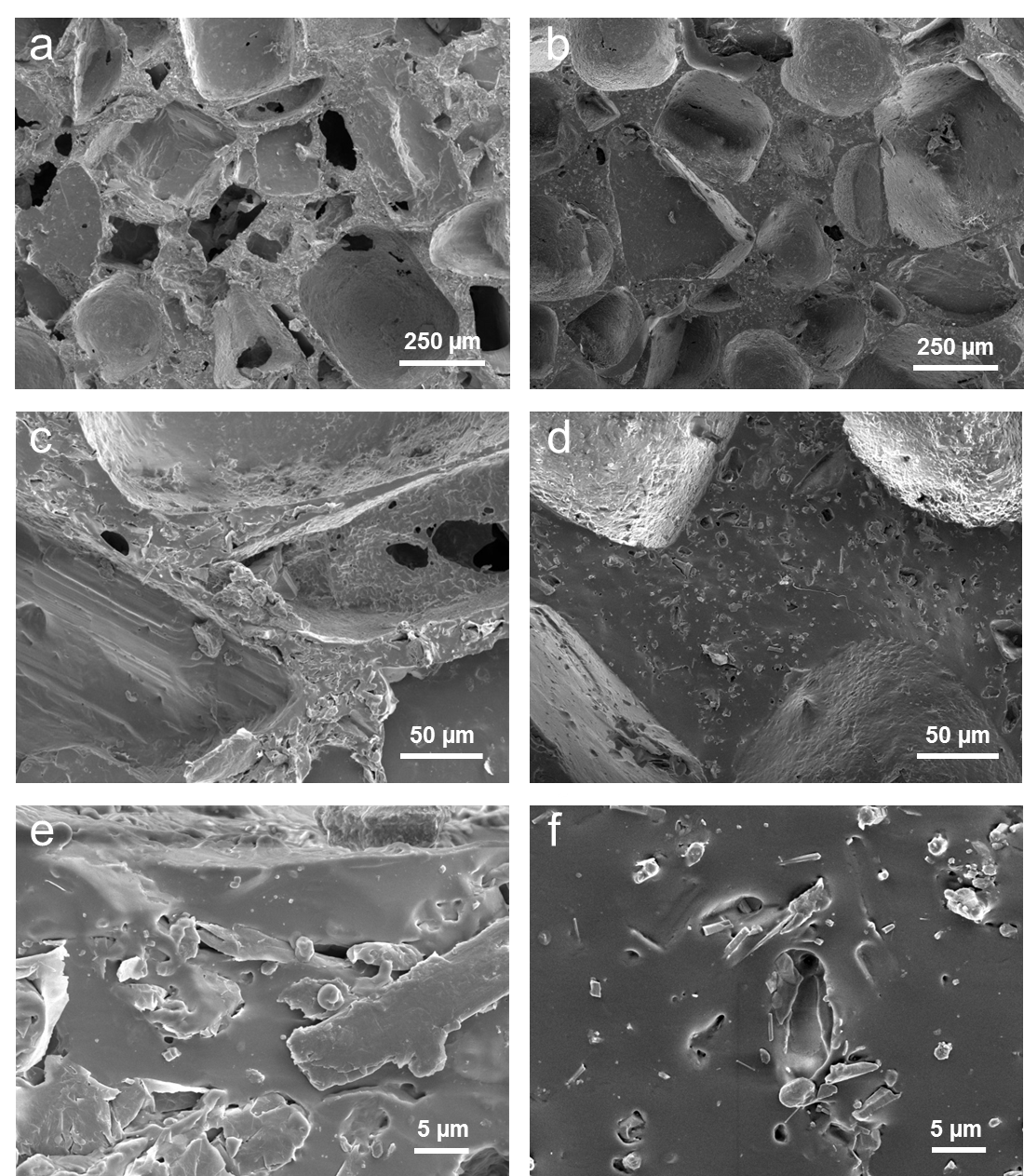


**Figure S1**. SEM image of the foam device (ZnO/graphene ratio is 0:1) at high magnification (a), medium magnification (c) and low magnification (e). SEM image of the foam device (ZnO/graphene ratio is 14:0) at low magnification (b), medium magnification (d) and high magnification (f).


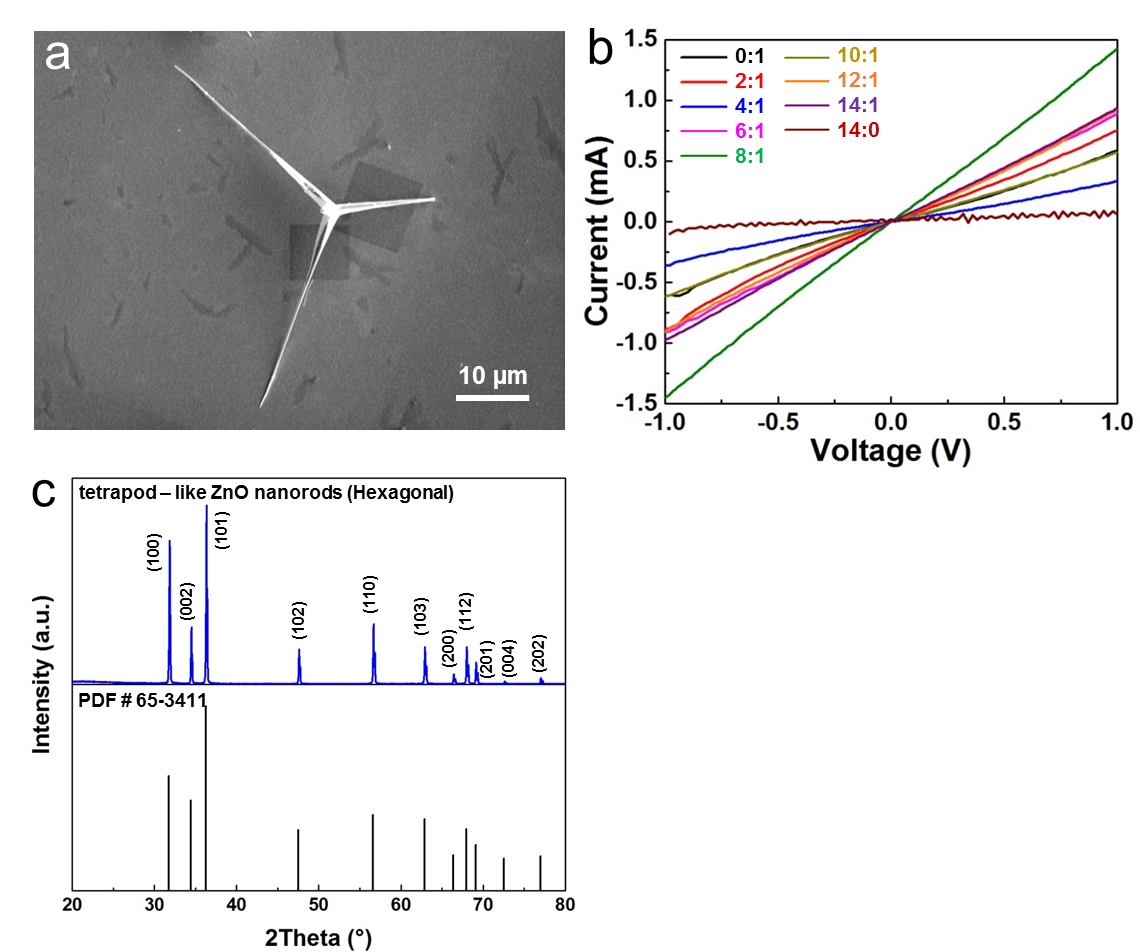


**Figure S2**. (a) SEM image of single tetrapod-like ZnO. (b) I-V curves of different ZnO/graphene ratio foam devices. (c) X-ray diffraction patterns of the tetrapod-like ZnO.


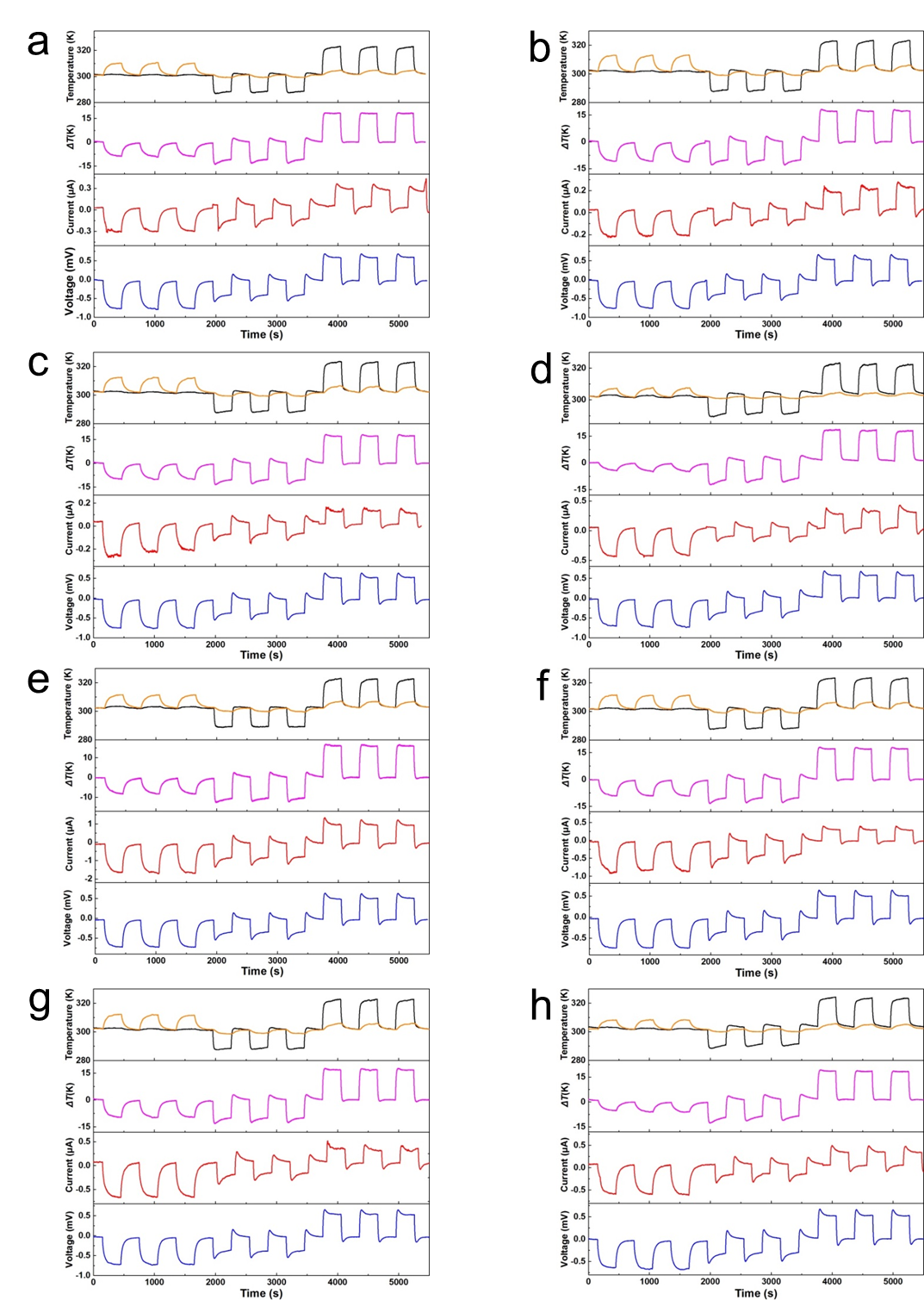


**Figure S3**. The output performances of different ZnO/graphene ratio foam-based devices. ZnO/graphene ratio is 0:1 (a), 2:1 (b), 4:1 (c), 6:1 (d), 8:1 (e), 10:1 (f), 12:1 (g), 14:1 (h).


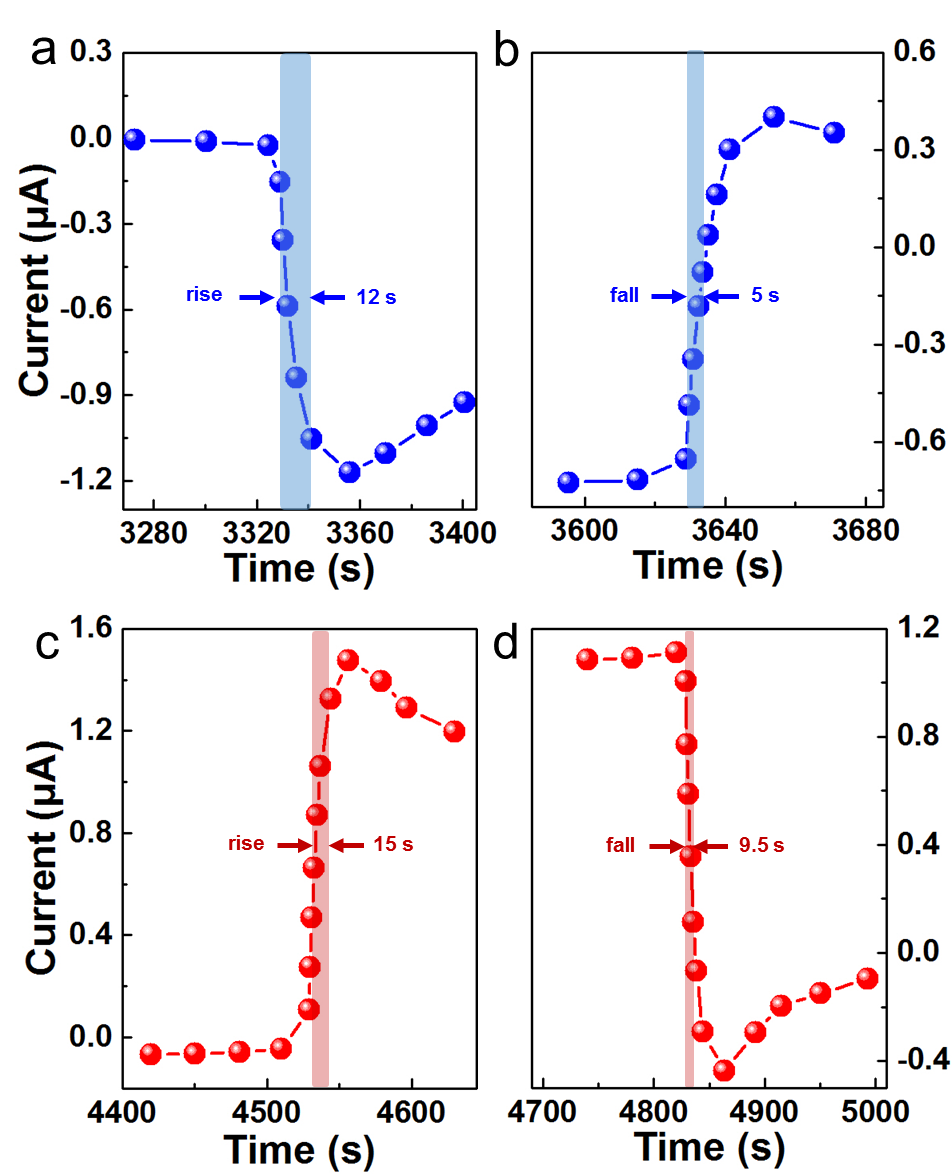


**Figure S4**. Response (a) and recovery time (b) of the device under cooling conduction. Response (c) and recovery time (d) of the device under heating conduction.


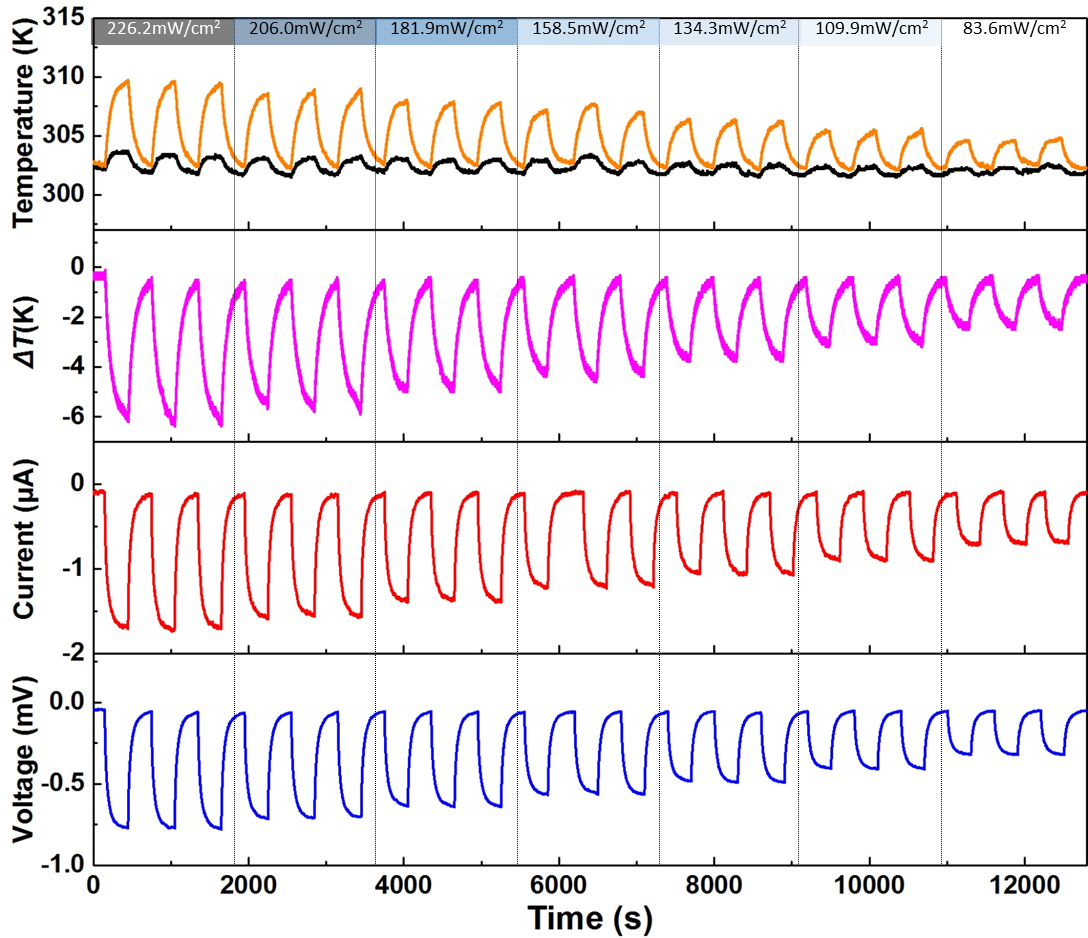


**Figure S5**. Output performance of the optimal device when the light intensity various from 226.2 mW/cm^2^ to 83.6 mW/cm^2^.


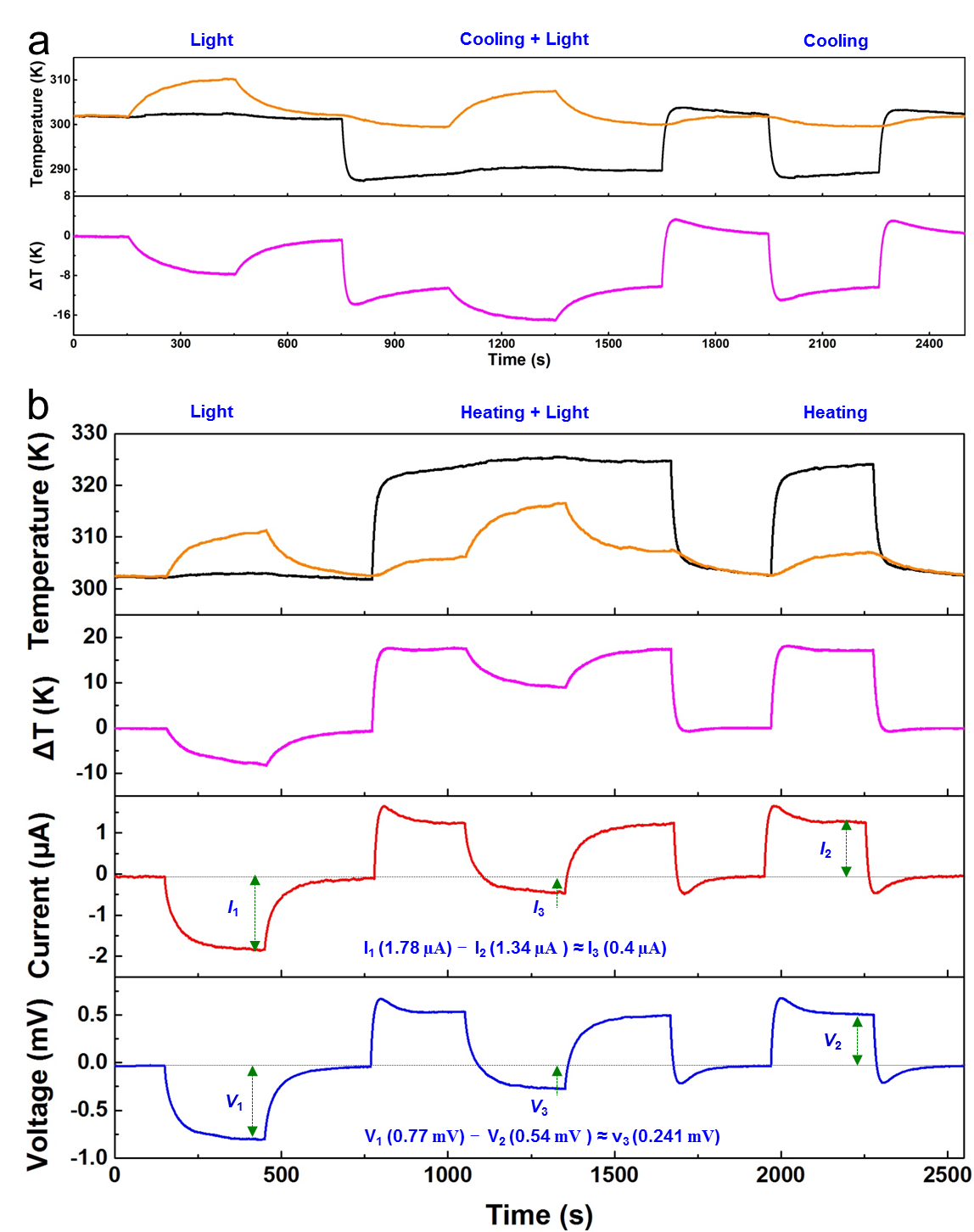


**Figure S6**. (a) Output performances of the ZnO-graphene nanocomposite foam-based device under lighting, light + cooling, and cooling condition. (b) Output performances of the ZnO-graphene nanocomposite foam-based device under lighting, light + heating, and heating condition


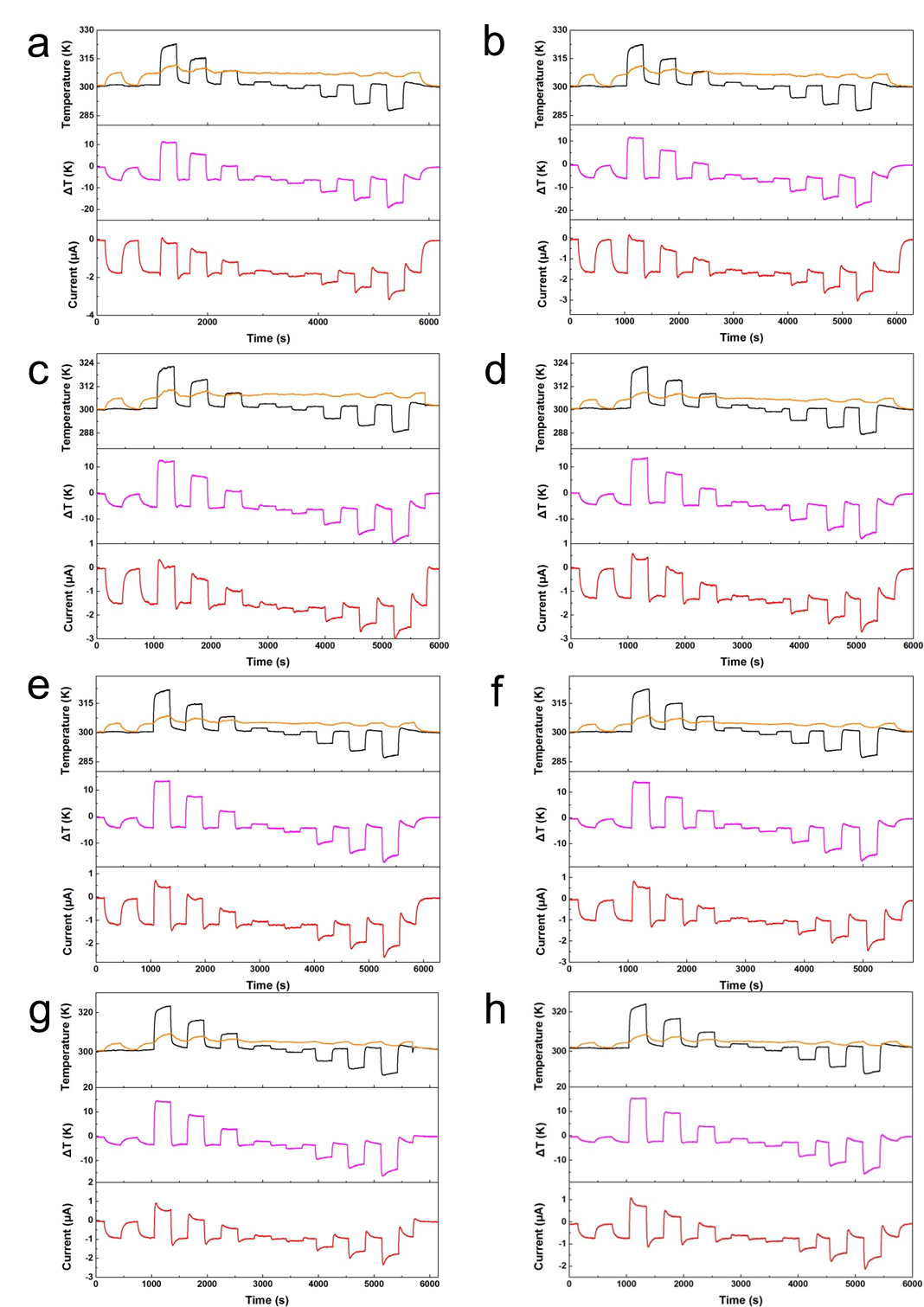


**Figure S7**. Output performances of the ZnO-graphene nanocomposite foam-based device at various temperature gradients when the intensity is (a) 247.8 mW/cm^2^, (b) 226.2 mW/cm^2^, (c) 206.0 mW/cm^2^, (d) 181.9 mW/cm^2^, (e) 158.5 mW/cm^2^, (f) 134.3 mW/cm^2^, (g) 109.9 mW/cm^2^, (h) 83.6 mW/cm^2^.


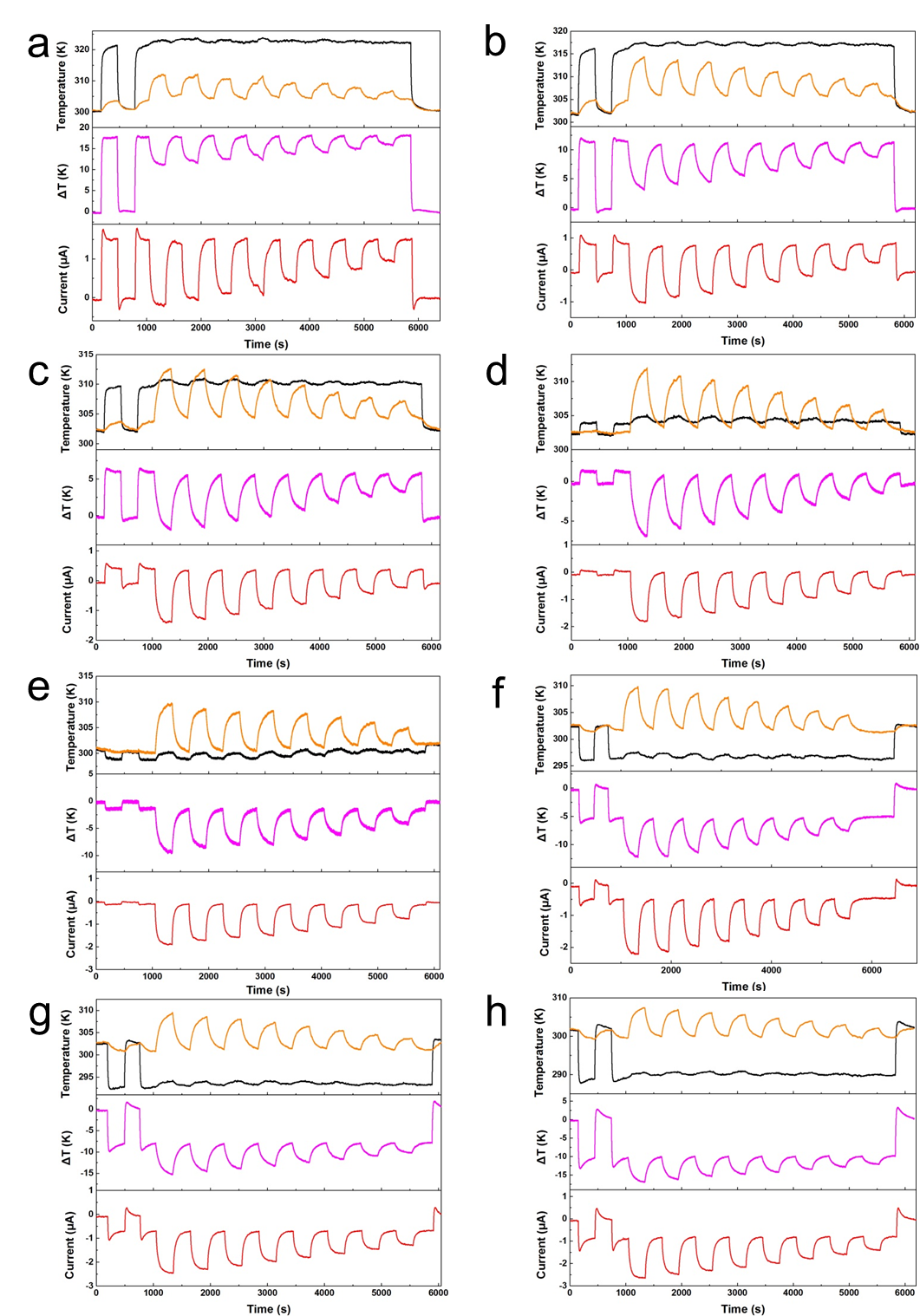


**Figure S8**. Output performances of the ZnO-graphene nanocomposite foam-based device under various light intensities when temperature difference is (a) 17.9 K, (b) 11.7 K, (c) 6.2 K, (d) 1.4 K, (e) -1.2 K, (f) -5.1 K, (g) -7.9 K, (h) -10.5 K.

**Table S1**. **The addition of T-ZnO and graphene with different ratio.**

| **Number** | **Ratio** | **T-ZnO (g)** | **Graphene (g)** |
| --- | --- | --- | --- |
| **1** | 0:1 | 0 | 0.09 |
| **2** | 2:1 | 0.18 | 0.09 |
| **3** | 4:1 | 0.36 | 0.09 |
| **4** | 6:1 | 0.54 | 0.09 |
| **5** | 8:1 | 0.72 | 0.09 |
| **6** | 10:1 | 0.9 | 0.09 |
| **7** | 12:1 | 1.08 | 0.09 |
| **8** | 14:1 | 1.26 | 0.09 |
| **9** | 1:0 | 1.26 | 0 |
